# Supplementary material for: Genetic and phylogenetic analysis of Chinese sacbrood virus isolates from Apis mellifera
Source: PeerJ. 2019 Nov 14;7:e8003. doi: 10.7717/peerj.8003 (PMC6858986; doi:10.7717/peerj.8003)
Supplement: Supplemental Information 1 — The highly conserved GPAGIGKS, QPVVVYDD, and KKIRGNPLIVILLCNH motifs were detected and marked with a red box in helicase domains A, B, and C, respectively [file peerj-07-8003-s001.pdf]

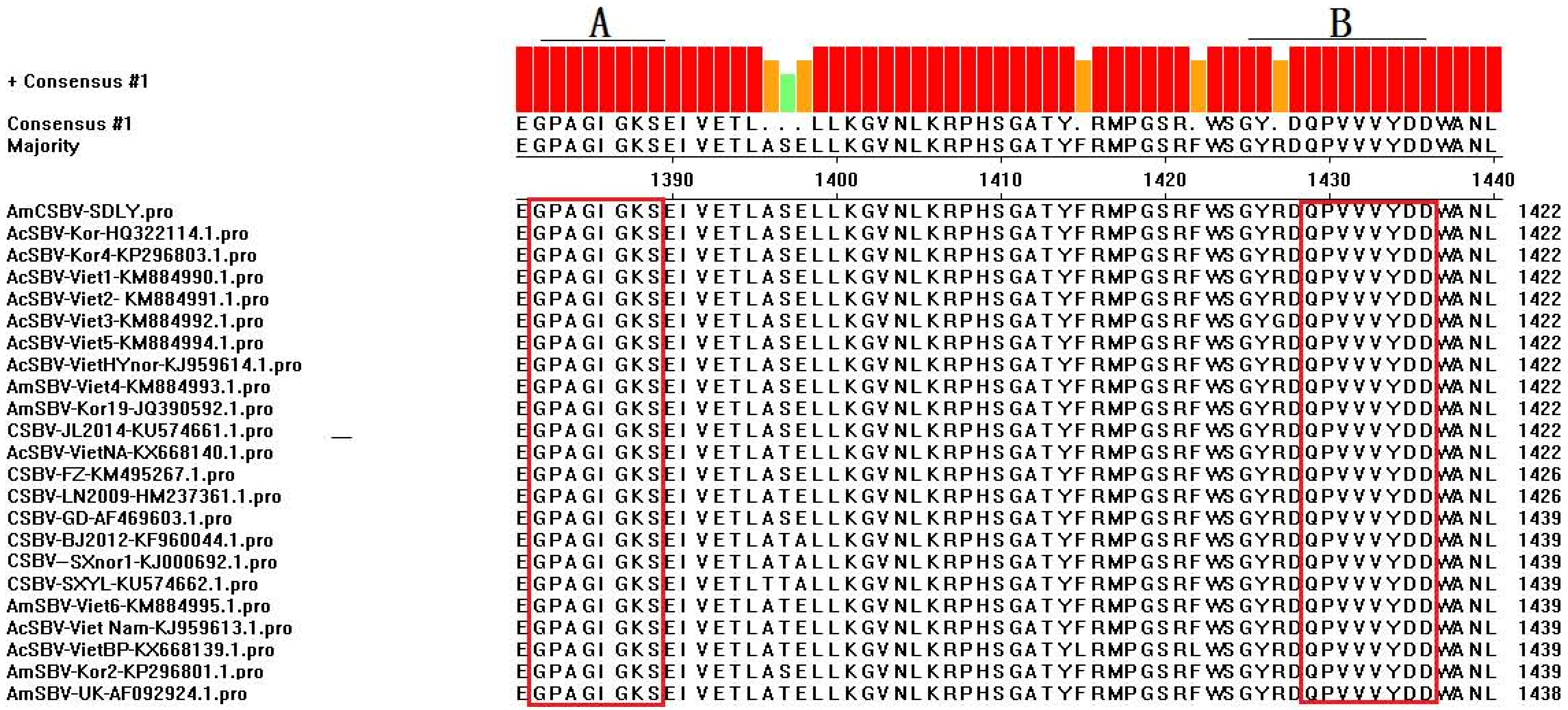
A
B
+ Consensus #1
Consensus #1 EGPAGIGKSEIVETL...LLKGVNLKRPHSGATY.RMPGSR.WSGY.DQPVVVYDDWANL
Majority EGPAGIGKSEIVETLASELLKGVNLKRPHSGATYFRMPGSRFWSGYRDQPVVVYDDWANL
1390
1400
1410
1420
1430
1440
AmCSBV-SDLY.pro EGPAGIGKSEIVETLASELLKGVNLKRPHSGATYFRMPGSRFWSGYRDQPVVVYDDWANL 1422
AcSBV-Kor-HQ322114.1.pro EGPAGIGKSEIVETLASELLKGVNLKRPHSGATYFRMPGSRFWSGYRDQPVVVYDDWANL 1422
AcSBV-Kor4-KP296803.1.pro EGPAGIGKSEIVETLASELLKGVNLKRPHSGATYFRMPGSRFWSGYRDQPVVVYDDWANL 1422
AcSBV-Viet1-KM884990.1.pro EGPAGIGKSEIVETLASELLKGVNLKRPHSGATYFRMPGSRFWSGYRDQPVVVYDDWANL 1422
AcSBV-Viet2- KM884991.1.pro EGPAGIGKSEIVETLASELLKGVNLKRPHSGATYFRMPGSRFWSGYRDQPVVVYDDWANL 1422
AcSBV-Viet3-KM884992.1.pro EGPAGIGKSEIVETLASELLKGVNLKRPHSGATYFRMPGSRFWSGYGDQPVVVYDDWANL 1422
AcSBV-Viet5-KM884994.1.pro EGPAGIGKSEIVETLASELLKGVNLKRPHSGATYFRMPGSRFWSGYRDQPVVVYDDWANL 1422
AcSBV-VietHYnor-KJ959614.1.pro EGPAGIGKSEIVETLASELLKGVNLKRPHSGATYFRMPGSRFWSGYRDQPVVVYDDWANL 1422
AmSBV-Viet4-KM884993.1.pro EGPAGIGKSEIVETLASELLKGVNLKRPHSGATYFRMPGSRFWSGYRDQPVVVYDDWANL 1422
AmSBV-Kor19-JQ390592.1.pro EGPAGIGKSEIVETLASELLKGVNLKRPHSGATYFRMPGSRFWSGYRDQPVVVYDDWANL 1422
CSBV-JL2014-KU574661.1.pro EGPAGIGKSEIVETLASELLKGVNLKRPHSGATYFRMPGSRFWSGYRDQPVVVYDDWANL 1422
AcSBV-VietNA-KX668140.1.pro EGPAGIGKSEIVETLATELLKGVNLKRPHSGATYFRMPGSRFWSGYRDQPVVVYDDWANL 1422
CSBV-FZ-KM495267.1.pro EGPAGIGKSEIVETLASELLKGVNLKRPHSGATYFRMPGSRFWSGYRDQPVVVYDDWANL 1426
CSBV-LN2009-HM237361.1.pro EGPAGIGKSEIVETLATELLKGVNLKRPHSGATYFRMPGSRFWSGYRDQPVVVYDDWANL 1426
CSBV-GD-AF469603.1.pro EGPAGIGKSEIVETLASELLKGVNLKRPHSGATYFRMPGSRFWSGYRDQPVVVYDDWANL 1439
CSBV-BJ2012-KF960044.1.pro EGPAGIGKSEIVETLATALLKGVNLKRPHSGATYFRMPGSRFWSGYRDQPVVVYDDWANL 1439
CSBV--SXnor1-KJ000692.1.pro EGPAGIGKSEIVETLATALLKGVNLKRPHSGATYFRMPGSRFWSGYRDQPVVVYDDWANL 1439
CSBV-SXYL-KU574662.1.pro EGPAGIGKSEIVETLTTALLKGVNLKRPHSGATYFRMPGSRFWSGYRDQPVVVYDDWANL 1439
AmSBV-Viet6-KM884995.1.pro EGPAGIGKSEIVETLATELLKGVNLKRPHSGATYFRMPGSRFWSGYRDQPVVVYDDWANL 1439
AcSBV-Viet Nam-KJ959613.1.pro EGPAGIGKSEIVETLATELLKGVNLKRPHSGATYFRMPGSRFWSGYRDQPVVVYDDWANL 1439
AcSBV-VietBP-KX668139.1.pro EGPAGIGKSEIVETLATELLKGVNLKRPHSGATYLRMPGSRLWSGYRDQPVVVYDDWANL 1439
AmSBV-Kor2-KP296801.1.pro EGPAGIGKSEIVETLASELLKGVNLKRPHSGATYFRMPGSRFWSGYRDQPVVVYDDWANL 1439
AmSBV-UK-AF092924.1.pro EGPAGIGKSEIVETLATELLKGVNLKRPHSGATYFRMPGSRFWSGYRDQPVVVYDDWANL 1438

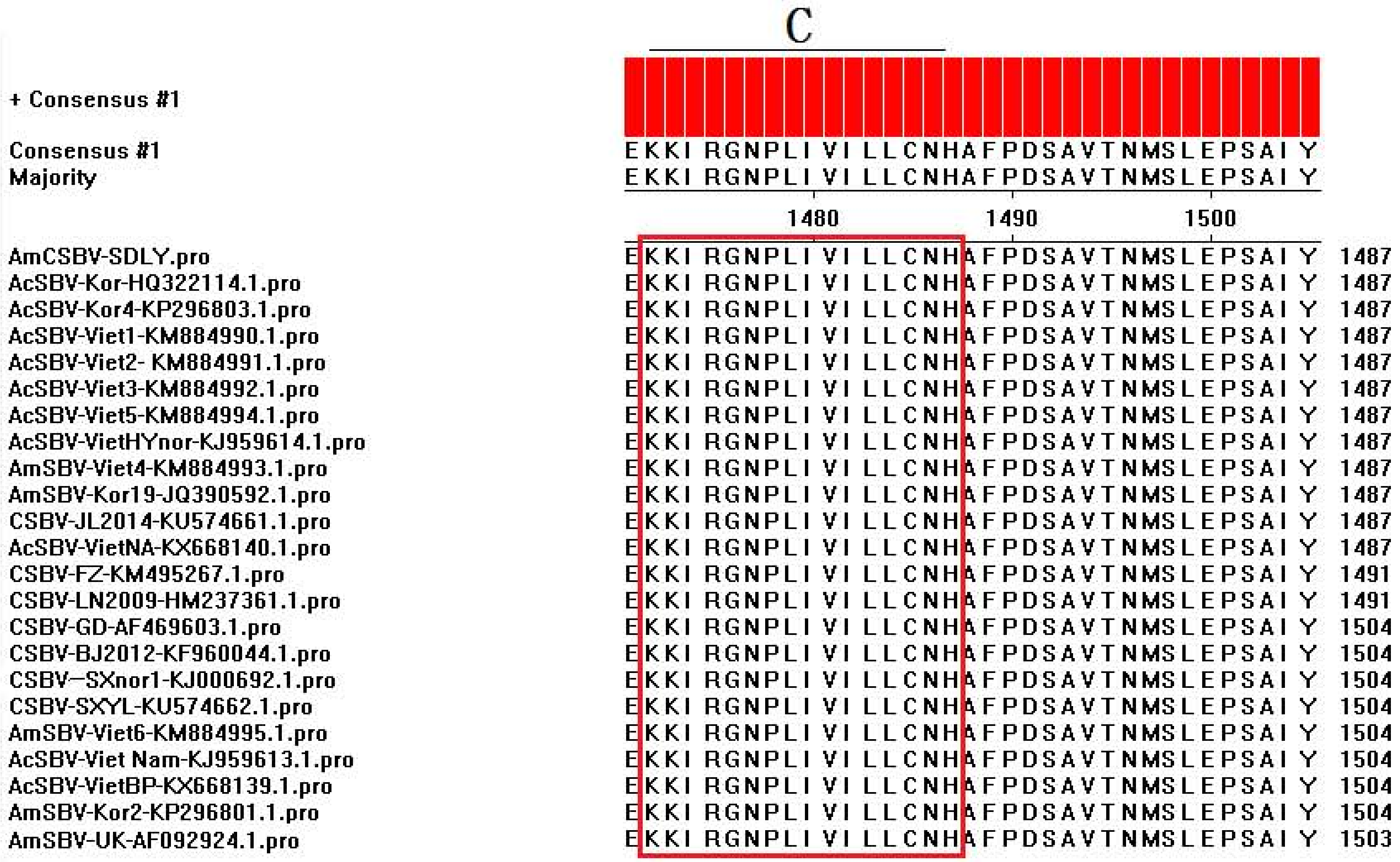
C
+ Consensus #1
Consensus #1 EKKIRGNPLIVILLCNHAFPDSAVTNMSLEPSAIY
Majority EKKIRGNPLIVILLCNHAFPDSAVTNMSLEPSAIY
1480 1490 1500
AmCSBV-SDLY.pro EKKIRGNPLIVILLCNHAFPDSAVTNMSLEPSAIY 1487
AcSBV-Kor-HQ322114.1.pro EKKIRGNPLIVILLCNHAFPDSAVTNMSLEPSAIY 1487
AcSBV-Kor4-KP296803.1.pro EKKIRGNPLIVILLCNHAFPDSAVTNMSLEPSAIY 1487
AcSBV-Viet1-KM884990.1.pro EKKIRGNPLIVILLCNHAFPDSAVTNMSLEPSAIY 1487
AcSBV-Viet2- KM884991.1.pro EKKIRGNPLIVILLCNHAFPDSAVTNMSLEPSAIY 1487
AcSBV-Viet3-KM884992.1.pro EKKIRGNPLIVILLCNHAFPDSAVTNMSLEPSAIY 1487
AcSBV-Viet5-KM884994.1.pro EKKIRGNPLIVILLCNHAFPDSAVTNMSLEPSAIY 1487
AcSBV-VietHYnor-KJ959614.1.pro EKKIRGNPLIVILLCNHAFPDSAVTNMSLEPSAIY 1487
AmSBV-Viet4-KM884993.1.pro EKKIRGNPLIVILLCNHAFPDSAVTNMSLEPSAIY 1487
AmSBV-Kor19-JQ390592.1.pro EKKIRGNPLIVILLCNHAFPDSAVTNMSLEPSAIY 1487
CSBV-JL2014-KU574661.1.pro EKKIRGNPLIVILLCNHAFPDSAVTNMSLEPSAIY 1487
AcSBV-VietNA-KX668140.1.pro EKKIRGNPLIVILLCNHAFPDSAVTNMSLEPSAIY 1487
CSBV-FZ-KM495267.1.pro EKKIRGNPLIVILLCNHAFPDSAVTNMSLEPSAIY 1491
CSBV-LN2009-HM237361.1.pro EKKIRGNPLIVILLCNHAFPDSAVTNMSLEPSAIY 1491
CSBV-GD-AF469603.1.pro EKKIRGNPLIVILLCNHAFPDSAVTNMSLEPSAIY 1504
CSBV-BJ2012-KF960044.1.pro EKKIRGNPLIVILLCNHAFPDSAVTNMSLEPSAIY 1504
CSBV—SXnor1-KJ000692.1.pro EKKIRGNPLIVILLCNHAFPDSAVTNMSLEPSAIY 1504
CSBV-SXYL-KU574662.1.pro EKKIRGNPLIVILLCNHAFPDSAVTNMSLEPSAIY 1504
AmSBV-Viet6-KM884995.1.pro EKKIRGNPLIVILLCNHAFPDSAVTNMSLEPSAIY 1504
AcSBV-Viet Nam-KJ959613.1.pro EKKIRGNPLIVILLCNHAFPDSAVTNMSLEPSAIY 1504
AcSBV-VietBP-KX668139.1.pro EKKIRGNPLIVILLCNHAFPDSAVTNMSLEPSAIY 1504
AmSBV-Kor2-KP296801.1.pro EKKIRGNPLIVILLCNHAFPDSAVTNMSLEPSAIY 1504
AmSBV-UK-AF092924.1.pro EKKIRGNPLIVILLCNHAFPDSAVTNMSLEPSAIY 1503
