# Supplementary material for: Genetic and phylogenetic analysis of Chinese sacbrood virus isolates from Apis mellifera
Source: PeerJ. 2019 Nov 14;7:e8003. doi: 10.7717/peerj.8003 (PMC6858986; doi:10.7717/peerj.8003)
Supplement: Supplemental Information 3 — Alignment of the amino acid sequences of RdRp from CSBVs. [file peerj-07-8003-s003.pdf]

```
                                                                                      I
                                                                               ________________
                               2470      2480      2490      2500      2510      2520      2530
AmCSBV-SDLY.pro                  2444 TSAGFPYVATEKKRKEDYIVFERNENEQPIGATIDPSVLEEMKRKSELRRQGVQPITPFIDTLKDERKLPEKVR 2517
AcSBV-Kor-HQ322114.1.pro              TSAGFPYVATEKKRKEDYIVFERNENEQPIGATIDPGVLEEMKRKSELRRQGVQPITPFIDTLKDERKLPEKVR 2517
AcSBV-Kor4-KP296803.1.pro             TSAGFPYVATEKKRKEDYIVFERNENEQPIGATIDPSVLEEMKRKSDLRRQGVQPITPFIDTLKDERKLPEKVR 2517
AcSBV-Viet1-KM884990.1.pro            TSAGFPYVATEKKRKEDYIVFERNENEQPIGATIDPSVLEEMKRKSELRRQGVQPITPFIDTLKDERKLPEKVR 2517
AcSBV-Viet2- KM884991.1.pro           TSAGFPYVATEKKRKEDYIVFERNENEQPIGATIDPSVLEEMKRKSELRRQGVQPITPFIDTLKDERKLPEKVR 2517
AcSBV-Viet3-KM884992.1.pro            TSAGFPYVATEKKRKEDYIVFERNENEQPIGATIDPSVLEEMKRKSELRRQGVQPITPFIDTLKDERKLPEKVR 2517
AcSBV-Viet5-KM884994.1.pro            TSAGFPYVATEKKRKEDYIVFERNENEQPIGATIDPSVLEEMKRKSDLRRQGVQPITPFIDTLKDERKLPEKVR 2517
AcSBV-VietHYnor-KJ959614.1.pro        TSAGFPYVATEKKRKEDYIVFERNENEQPIGATIDPSVLEEMKRKSDLRRQGVQPITPFIDTLKDERKLPEKVR 2517
AmSBV-Viet4-KM884993.1.pro            TSAGFPYVATEKKRKEDYIVFERNENEQPIGATIDPSVLEEMKRKSELRRQGVQPITPFIDTLKDERKLPEKVR 2517
AmSBV-Kor19-JQ390592.1.pro            TSAGFPYVATEKKRKEDYIVFERNENEQPIGATIDPGVLEEMKRKSELRRQGVQPITPFIDTLKDERKLPEKVR 2517
CSBV-JL2014-KU574661.1.pro            TSAGFPYVATDKKRKEDYIVFERNENEQPIGATIDPSVLEEMKRKSELRRQGVQPITPFIDTLKDERKLPEKVR 2517
AcSBV-VietNA-KX668140.1.pro           TSAGFPYVATEKKRKEDYIVFERNENEQPIGATIDPSVLEEMKRKSDLRRQGVQPITPFIDTLKDERKLPEKVR 2517
CSBV-FZ-KM495267.1.pro                TSAGFPYVATEKKRKEDYIVFERNENEQPIGATIDPSVLEEMKRKSELRRQGVQPITPFIDTFKDERKLPEKVR 2522
CSBV-LN2009-HM237361.1.pro            TSAGFPYVATDKKRKEDYIVFERNENEQPIGATIDPSVLEEMKRKSELRRQGVQPITPFIDTLKDERKLPEKVR 2522
CSBV-GD-AF469603.1.pro                TSAGFPYVATEKKRKEDYIVFERNENEQPIGATIDPGVLEEMKRKSELRRQGVQPITPFIDTLKDERKLPEKVR 2535
CSBV-BJ2012-KF960044.1.pro            TSAGFPYVATEKKRKEDYIVFERNENEQPIGATIDPSVLEEMKRKSELRRQGVQPITPFIDTLKDERKLPEKVR 2535
CSBV--SXnor1-KJ000692.1.pro           TSAGFPYVATEKKRKEDYIVFERNENEQPIGAIIDPSVLEEMKRKSELRRQGVQPITPFIDTLKDERKLPEKVR 2535
CSBV-SXYL-KU574662.1.pro              TSAGFPYVATEKKRKEDYIVFERNENEQPIGAAIDPGVLGEMKRKSELRRQGVQPITPFIDTLKDERKLPEKVR 2534
AmSBV-Viet6-KM884995.1.pro            TSAGFPYVATEKKRKEDYIVFERNENEQPIGGTIDPGVLEEMKRKSELRRQGVQPITPFIDTLKDERKLPEKVR 2534
AcSBV-Viet Nam-KJ959613.1.pro         TSAGFPYVATEKKRKEDYIVFERNENEQPIGATIDPGVLEEMKRKSELRRQGVQPITPFIDTLKDERKLPEKVR 2534
AcSBV-VietBP-KX668139.1.pro           TSAGFPSVATEKKRKEDYIVFERNENEQPIGATIDPGVLEEMKRKSELRRQGVQPITPFIDTLKDERKLPEKVR 2534
AmSBV-Kor2-KP296801.1.pro             TSAGFPYVATEKKRKEDYIVFERNENEQPIGATIDPSVLEEMKRKSDLRRQGVQPITPFIDTLKDERKLPEKVR 2534
AmSBV-UK-AF092924.1.pro               TSAGFPYVATEKKRKEDYIVFERNENEQPIGATIDPGVLEEMKRKSELRKQGVQPITPFIDTLKDERKLPEKVR 2533
```

```
                                              II                                  III
                                   2540      2550      2560      2570      2580      2590      2600
AmCSBV-SDLY.pro                    KYGGTRVFCNPPIDYIVSMRQYYMHFVAAFMEQRFKLMHAVGINVQSTEWTLLASKLLAKGNNIC  2582
AcSBV-Kor-HQ322114.1.pro           KYGGTRVFCNPPIDYIVSMRQYYMHFVAAFMEQRFKLMHAVGINVQSTEWTLLASKLLAKGNNIC  2582
AcSBV-Kor4-KP296803.1.pro          KYGGTRVFCNPPIDYIVSMRQYYMHFVAAFMEQRFKLMHAVGINVQSTEWTLLASKLLAKGNNIC  2582
AcSBV-Viet1-KM884990.1.pro         KYGGTRVFCNPPIDYIVSMRQYYMHFVAAFMEQRFKLMHAVGINVQSTEWTLLASKLLAKGNNIC  2582
AcSBV-Viet2- KM884991.1.pro        KYGGTRVFCNPPIDYIVSMRQYYMHFVAAFMEQRFKLMHAVGINVQSTEWTLLASKLLAKGNNIC  2582
AcSBV-Viet3-KM884992.1.pro         KYGGTRVFCNPPIDYIVSMRQYYMHFVAAFMEQRFKLMHAVGINVQSTEWTLLASKLLAKGNNIC  2582
AcSBV-Viet5-KM884994.1.pro         KYGGTRVFCNPPIDYIVSMRQYYMHFVAAFMEQRFKLMHAVGINVQSTEWTLLASKLLAKGNNIC  2582
AcSBV-VietHYnor-KJ959614.1.pro     KYGGTRVFCNPPIDYIVSMRQYYMHFVAAFMEQRFKLMHAVGINVQSTEWTLLASKLLAKGNNIC  2582
AmSBV-Viet4-KM884993.1.pro         KYGGTRVFCNPPIDYIVSMRQYYMHFVAAFMEQRFKLMHAVGINVQSTEWTLLASKLLAKGNNIC  2582
AmSBV-Kor19-JQ390592.1.pro         KYGGTRVFCNPPIDYTVSMRQYYMHFVAAFMEQRFKLMHAVGINVQSTEWTLLASKLLAKGNNIC  2582
CSBV-JL2014-KU574661.1.pro         KYGGTRVFCNPPIDYIVSMRQYYMHFVAAFMEQRFKLMHAVGINVQSTEWTLLASKLLAKGNNIC  2582
AcSBV-VietNA-KX668140.1.pro        KYGGTRVFCNPPIDYIVSMRQYYMHFVAAFMEQRFKLMHAVGINVQSTEWTLLASKLLAKGNNIC  2582
CSBV-FZ-KM495267.1.pro             KYGGTRVFCNPPIDYIVSMRQYYMHFVAAFMEQRFKLMHAVGINVQSTEWTLLASKLLAKGNNIC  2587
CSBV-LN2009-HM237361.1.pro         KYGGTRVFCNPPIDYIVSMRQYYMHFVAAFMEQRFKLMHAVGINVQSTEWTLLASKLLAKGNNIC  2587
CSBV-GD-AF469603.1.pro             KYGGTRVFCNPPIDYIVSMRQYYMHFVAAFMEQRFKLMHAVGINVQSTEWTLLASKLLAKGNNIC  2600
CSBV-BJ2012-KF960044.1.pro         KYGGTRVFCNPPIDYIVSMRQHYMHFVAAFMEQRFKLMHAVGINVQSTEWTLLASKLLAKGNNIC  2600
CSBV—SXnor1-KJ000692.1.pro         KYGGTRVFCNPPIDYIVSMRQHYMHFVAAFMEQRFKLMHAVGINVQSTEWTLLASKLLAKGNNIC  2600
CSBV-SXYL-KU574662.1.pro           KYGGTRVFCNPPIDYIVSMRQHYMHFVAAFMEYRFKLMHAVGINVQSTEWTLLASKLLAKGNNIC  2599
AmSBV-Viet6-KM884995.1.pro         KYGGTRVFCNPSIDYIVSMRQYYMHFVAAFMEQRFKLMHAVGINVQSTEWTLLASKLLAKGNNIC  2599
AcSBV-Viet Nam-KJ959613.1.pro      KYGGTRVFCNPSIDYIVSMRQYYMHFVAAFMEQRFKLMHAVGINVQSTEWTLLASKLLAKGNNIC  2599
AcSBV-VietBP-KX668139.1.pro        KYGGTRVFCYPSIDYIVSMRQYYMHFVAAYMELRFKLMHAVGINVQSTEWTLLASKLLAKGNNIC  2599
AmSBV-Kor2-KP296801.1.pro          KYGGTRVFCNPPIDYIVSMRQYYMHFVAAFMEQRFKLMHAVGIKVQSTEWTLLASKLLAKGNNIC  2599
AmSBV-UK-AF092924.1.pro            KYGGTRVFCNPPIDYIVSMRQYYMHFVAAFMEQRFKLMHAVGINVQSTEWTLLASKLLAKGNNIC  2598
```

```
                                          IV
                                            2610      2620      2630      2640      2650      2660
AmCSBV-SDLY.pro                        TIDYSNFGPGFNAQIAKAAMELMVRWTMEHVEGVNEIEAHTLLHECLNSVHLVSNTLYQQKCGSP  2647
AcSBV-Kor-HQ322114.1.pro               TIDYSNFGPGFNAQIAKAAMELMVRWTMEHVEGVNEIEANTLLHECLNSVHLVSNTLYQQKCGSP  2647
AcSBV-Kor4-KP296803.1.pro              TIDYSNFGPGFNAQIAKAAMELMVRWTMEHVEGVNEIEAHTLLHECLNSVHLVSNTLYQQKCGSP  2647
AcSBV-Viet1-KM884990.1.pro             TIDYSNFGPGFNAQIAKAAMELMVRWTMEHVEGVNEIEAHTLLHECLNSVHLVSNTLYQQKCGSP  2647
AcSBV-Viet2- KM884991.1.pro            TIDYSNFGPGFNAQIAKAAMELMVRWTMEHVEGVNEIEAHTLLHECLNSVHLVSNTLYQQKCGSP  2647
AcSBV-Viet3-KM884992.1.pro             TIDYSNFGPGFNAQIAKAAMELMVRWTMEHVEGVNEIEAHTLLHECLNSVHLVSNTLYQQKCGSP  2647
AcSBV-Viet5-KM884994.1.pro             TIDYSNFGPGFNAQIAKAAMELVVRWTMEHVEGVNEIEAHTLLHECLNSVHLVSNTLYQQKCGSP  2647
AcSBV-VietHYnor-KJ959614.1.pro         TIDYSNFGPGFNAQIAKAAMELMVRWTMEHVEGVNEIEAHTLLHECLNSVHLVSNTLYQQKCGSP  2647
AmSBV-Viet4-KM884993.1.pro             TIDYSNFGPGFNAQIAKAAMELMVRWTMEHVEGVNEIEAHTLLHECLNSVHLVSNTLYQQKCGSP  2647
AmSBV-Kor19-JQ390592.1.pro             TIDYSNFGPGFNAQIAKAAMELVVRWTMEHVEGVNEIEANTLLHECLNSVHLVSNTLYQQKCGSP  2647
CSBV-JL2014-KU574661.1.pro             TIDYSNFGPGFNAQIAKAAMELMVRWTMEHVEGVNETEAHTLLHECLNSVHLVSNTLYQQKCGSP  2647
AcSBV-VietNA-KX668140.1.pro            TIDYSNFGPGFNAQIAKAAMELMVRWTMEHVEGVNEIEAYTLLHECLNSVHLVSNTLYQQKCGSP  2647
CSBV-FZ-KM495267.1.pro                 TIDYSNFGPGFNAQIAKAAMELMVRWTMEHVEGVNEIEAHTLLHECLNSVHLVSNTLYQQKCGSP  2652
CSBV-LN2009-HM237361.1.pro             TIDYSNFGPGFNAQIAKAAMELMVRWTMEHVEGVNETEAHTLLHECLNSVHLVSNTLYQQKCGSP  2652
CSBV-GD-AF469603.1.pro                 TIDYSNFGPGFNAQIAKAAMELMVRWTMEHVEGVNEIEAYTLLHECLNSVHLVSNTLYQQKCGSP  2665
CSBV-BJ2012-KF960044.1.pro             TIDYSNFGPGFNAQIAKAAMELMVRWTMEHVEGVNEIEAHTLLHECLNSVHLVSNTLYQQKCGSP  2665
CSBV--SXnor1-KJ000692.1.pro            TIDYSNFGPGFNAQIAKAAMELMVRWTMEHVEGVNEIEAHTLLHECLNSVHLVSNTLYQQKCGSP  2665
CSBV-SXYL-KU574662.1.pro               TIDYSNFGPGFNAQIAKAAMELMVRWTMEHVEGVNEIEAHTLLHECLNSYHLVSNTLYQQKCGSP  2664
AmSBV-Viet6-KM884995.1.pro             TIDYSNFGPGFNAQIAKAAMDLMVRWTMEHVEGVNELEAYTLLHECLNSVHLVSNTLYQQKCGSP  2664
AcSBV-Viet Nam-KJ959613.1.pro          TIDYSNFGPGFNAQIAKAAMDLMVRWTMEHVEGVNELEAYTLLHECLNSVHLVSNTLYQQKCGSP  2664
AcSBV-VietBP-KX668139.1.pro            TIDYSNFGPGFNAQLAKAAMDLMVRWTMEHVEGVNELVAYTLLHECLNSVQLVSNTLYQQKCGSP  2664
AmSBV-Kor2-KP296801.1.pro              TIDYSNFGPGFNAQIAKAAMELMVRWTMEHVEGVNEIEAHTLLHECLNSVHLVSNTLYQQKCGSP  2664
AmSBV-UK-AF092924.1.pro                TIDYSNFGPGFNAQIAKAAMELMVRWTMEHVEGVNEIEAYTLLHECLNSVHLVSNTLYQQKCGSP  2663
```

```
                                   V                        VI
                                  2670  2680  2690  2700  2710  2720  2730
AmCSBV-SDLY.pro                    SGAPITVVINTLVNILYIFVAWETLVGSKERGQTWESFKQNVELFCYGDDLIMSVTDIYKDTFNA  2712
AcSBV-Kor-HQ322114.1.pro           SGAPITVVINTLVNILYIFVAWETLVGSKERGQMWEIFKQNVELFCYGDDLIMSVTDKYKDTFNA  2712
AcSBV-Kor4-KP296803.1.pro          SGAPITVVINTLVNILYIFVAWETLVGSKERGQTWESFKLNVELFCYGDDLIMSVTDKYKDIFNA  2712
AcSBV-Viet1-KM884990.1.pro         SGAPITVVINTLVNILYIFVAWETLVGSKERGQTWESFKQNVELFCYGDDLIMSVTEKYKDTFNA  2712
AcSBV-Viet2- KM884991.1.pro        SGAPITVVINTLVNILYIFVAWETLVGSKERGQTWESFKQNVELFCYGDDLIMSVTEKYKDTFNA  2712
AcSBV-Viet3-KM884992.1.pro         SGAPITVVINTLVNILYIFVAWETLVGSKERGQTWESFKQNVELFCYGDDLIMSVTEKYKDTFNA  2712
AcSBV-Viet5-KM884994.1.pro         SGAPITVVINTLVNILYIFVAWETLVGSKERGQTWESFKLNVELFCYGDDLIMSVTDKYKDIFNA  2712
AcSBV-VietHYnor-KJ959614.1.pro     SGAPITVVINTLVNILYIFVAWETLVGSKERGQTWESFKLNVELFCYGDDLIMSVTDKYKDIFNA  2712
AmSBV-Viet4-KM884993.1.pro         SGAPITVVINTLVNILYIFVAWETLVGSKERGQTWESFKQNVELFCYGDDLIMSVTEKYKDTFNA  2712
AmSBV-Kor19-JQ390592.1.pro         SGAPITVVINTLVNILYIFVAWETLVGSKERGQMWEIFKQNVELFCYGDDLIMSVTDKYKDTFNA  2712
CSBV-JL2014-KU574661.1.pro         SGAPITVVINTLVNILYIFVAWEMLVGSKEKGQTWESFKQNVELFCYGDDLIMSVTDKYKDAFNA  2712
AcSBV-VietNA-KX668140.1.pro        SGAPITVVINTLVNILYIFVAWETLVGSRERGQTWESFRQNVELFCYGDDLIMSVTDKYKDTFNA  2712
CSBV-FZ-KM495267.1.pro             SGAPITVVINTLVNILYIFVAWETLVGSKERGQTWESFKQNVELFCYGDDLIMSVTDKYKDTFNA  2717
CSBV-LN2009-HM237361.1.pro         SGAPITVVINTLVNILYIFVAWEMLVGSKEKGQTWESFKQNVELFCYGDDLIMSVTDKYKDAFNA  2717
CSBV-GD-AF469603.1.pro             SGAPITVVINTLVNILYIFVAWETLVGSKERGQTWESFKQNVELFCYGDDLIMSVTDKYKDIFNA  2730
CSBV-BJ2012-KF960044.1.pro         SGAPITVVINTLVNILYIFVAWETQVGSKEKGQTWESFKQNIELFCYGDDLIMSVTDKYKETFNA  2730
CSBV--SXnor1-KJ000692.1.pro        SGAPITVVINTLVNILYIFVAWETLVGSKERGQTWESFKQNIELFCYGDDLIMSVTDKYKETFNA  2730
CSBV-SXYL-KU574662.1.pro           SGAPITVVINTLVNILYIFVAWETLVGSKERGQTWESFKQNIELFCYGDDLIMSVTDKYKHTFNA  2729
AmSBV-Viet6-KM884995.1.pro         SGAPITVVINTLVNILYIFVAWETLVGSRERGQTWESFKQNVELFCYGDDLIMSVTDKYKDNFNA  2729
AcSBV-Viet Nam-KJ959613.1.pro      SGAPITVVINTLVNILYIFVAWETLVGSRERGQTWESFKQNVELFCYGDDLIMSVTDKYKDNFNA  2729
AcSBV-VietBP-KX668139.1.pro        SGAPITVVINTLVNILYIFVAWETLVGSRERGQTWESFKQNVELFCYGDDLIMSVTDKYKDNFNA  2729
AmSBV-Kor2-KP296801.1.pro          SGAPITVVINTLVNILYIFVAWETLVGSKERGQTWESFKLNVELFCYGDDLIMSVTDKYKDTFNA  2729
AmSBV-UK-AF092924.1.pro            SGAPITVVINTLVNILYIFVAWETLVGSKERGQTWESFKQNVELFCYGDDLIMSVTDKYKDVFNA  2728
```

```
                                                      VII
                                          2740       2750       2760       2770       2780       2790
AmCSBV-SDLY.pro                    LTISQFLAQYGIVATDANKGEKVEAYTTLLNSTFLKHGFRLHEVYPHCGQSALAWSSIND 2773
AcSBV-Kor-HQ322114.1.pro           LTISQFLAQYGIVATDANKGEEVKAYTTLLNSTFLKHGFRPHEVYPHLWQSALAWSSIND 2773
AcSBV-Kor4-KP296803.1.pro          LTISQFLAHYGIVATDANKGEKVEAYSTLVNSTFLKHGFRPHEVYPHLWQSALAWSSIND 2773
AcSBV-Viet1-KM884990.1.pro         LTISQFLAQYGIVATDANKGEKVEAYATLVNSTFLKHGFRPHEVYPHLWQSALAWNSIND 2773
AcSBV-Viet2- KM884991.1.pro        LTISQFLAQYGIVATDANKGEKVEAYATLVNSTFLKHGFRPHEVYPHLWQSALAWNSIND 2773
AcSBV-Viet3-KM884992.1.pro         LTISQFLAQYGIVATDANKGEKVEAYATLVNSTFLKHGFRPHEVYPHLWQSALAWNSIND 2773
AcSBV-Viet5-KM884994.1.pro         LTISQFLAHYGIVATDANKGEKVEAYSTLVNSTFLKHGFRPHEVYPHLWQSALAWGSIND 2773
AcSBV-VietHYnor-KJ959614.1.pro     LTISQFLAHYGIVATDANKGEKVEAYSTLVNSTFLKHGFRPHEVYPHLWQSALAWGSIND 2773
AmSBV-Viet4-KM884993.1.pro         LTISQFLAQYGIVATDANKGEKVEAYATLVNSTFLKHGFRPHEVYPHLWQSALAWSSIND 2773
AmSBV-Kor19-JQ390592.1.pro         LTMSQFLAQYGIVATDANKGEEVKAYTTLLNSTFLKHGFRPHEVYPHLWQSALAWSSIND 2773
CSBV-JL2014-KU574661.1.pro         LTISQFLAQYGIVATDANKGEEVEAYTTLLNSTFLKHGFRPHEVYPHLWQSALAWNSIND 2773
AcSBV-VietNA-KX668140.1.pro        LTISQFLAQYGIVATDANKGEEVEAYTTLINSTFLKHGFRPHEVYPHLWQSALVWSSIND 2773
CSBV-FZ-KM495267.1.pro             LTISQFLAQYGIVATDANKGEKVEAYTTLLNSTFLKHGFRPHEVYPHLWQSALAWSSIND 2778
CSBV-LN2009-HM237361.1.pro         LTISQFLAQYGIVATDANKGEEVEAYTTLLNSTFLKHGFRPHEVYPHLWQSALAWNSIND 2778
CSBV-GD-AF469603.1.pro             LTISQFLAQYGIVATDANKGEEVEAYTTLLNSTFLKHGFRPHEVYPHLWQSALAWSSIND 2791
CSBV-BJ2012-KF960044.1.pro         LTISQFLAQYGIVATDANKGEEVEAYTTLLDSTFLKHGFRPHEVYPHLWQSALAWSSIND 2791
CSBV—SXnor1-KJ000692.1.pro         LTISQFLAQYGIVATDANKGEEVEAYTTLLDSTFLKHGFRPHEVYPHLWQSALAWSSIND 2791
CSBV-SXYL-KU574662.1.pro           LTISQFLAQYGIVATDANKGEEVEAYTTLLDSTFLKHGFRPHEVYPHLWQSALAWGSIND 2790
AmSBV-Viet6-KM884995.1.pro         LTISQFLAQYGIVATDANKGEEVEAYTTLLNSTFLKHGFRPHEVYPHLWQSALAWSSIND 2790
AcSBV-Viet Nam-KJ959613.1.pro      LTISQFLAQYGIVATDANKGEEVEAYTTLLNSTFLKHGFRPHEVYPHLWQSALAWSSIND 2790
AcSBV-VietBP-KX668139.1.pro        LTISQFLAQYGIVATDANKGEEVEAYTTLLNSTFLKHGFRPHEVYPHLWQSALAWSSIND 2790
AmSBV-Kor2-KP296801.1.pro          LTISRFLAQYGIVATDANKGEEVKAYTTLLNSTFLKHGFRPHEVYPHLWQSALAWSSIND 2790
AmSBV-UK-AF092924.1.pro            LTISQFLAQYGIVATDANKGDEVEAYTTLLNSTFLKHGFHPHEVYPHLWQSALAWSSIND 2789
```

VIII

```
                                          2800      2810      2820      2830      2840
AmCSBV-SDLY.pro                        TTQWWECADLKLATRENCRAALYQAHGHGSVVYNRFKQQVNKALIKRKIQPIALSW  2829
AcSBV-Kor-HQ322114.1.pro               TTQWWECADLKLATRENCRAALYQAHGHGSVVYNRFKQQVNKALVKRKIQPIALSW  2829
AcSBV-Kor4-KP296803.1.pro              TTQWWECADLKLATRENCRAALYQAHGHGSVVYNRFKQQVNKALIKRKIQPIALSW  2829
AcSBV-Viet1-KM884990.1.pro             TTQWWECADLKLATRENCRAALYQAHGHGSVVYNRFKQQVNKALIKRKIQPIALSW  2829
AcSBV-Viet2- KM884991.1.pro            TTQWWECADLKLATRENCRAALYQAHGHGSVVYNRFKQQVNKALIKRKIQPIALSW  2829
AcSBV-Viet3-KM884992.1.pro             TTQWWECADLKLATRENCRAALYQAHGHGSVVYNRFKQQVNKALIKRKIQPIALSW  2829
AcSBV-Viet5-KM884994.1.pro             TTQWWECADLKLATRENCRAAVYQAHGHGSVVYNRFKQQVIKALIKRKIQPIALSW  2829
AcSBV-VietHYnor-KJ959614.1.pro         TTQWWECADLKLATRENCRAALYQAHGHGSVVYNRFKQQVNKALIKRKIQPIALSW  2829
AmSBV-Viet4-KM884993.1.pro             TTQWWECADLKLATRENCRAALYQAHGHGSVVYNRFKQQVNKALIKRKIQPIALSW  2829
AmSBV-Kor19-JQ390592.1.pro             TTQWWECADLKLATRENCRAALYQAHGHGSVVYNRFKQQVNKALIKRKIQPIALSW  2829
CSBV-JL2014-KU574661.1.pro             TTQWWECADLKLATRENCRAALYQAHGHGSVVYNRFKQQVNKALVKRKIQPIALSW  2829
AcSBV-VietNA-KX668140.1.pro            TTQWWECADLKLATRENCRAALYQAHGHGSIVYNRFKQQVNKALIKRKIQPIALSW  2829
CSBV-FZ-KM495267.1.pro                 TTQWWECADLKLATRENCRAALYQAHGHGSVVYNRFKQQVNKALIKRKIQPIALSW  2834
CSBV-LN2009-HM237361.1.pro             TTQWWECADLKLATRENCRAALYQAHGHGSVVYNRFKQQVNKALIKRKIQPIALSW  2834
CSBV-GD-AF469603.1.pro                 TTQWWECADLKLATRENCRAALYQAHGHGSVVYNRFKQQVNKALIKRKIQPIALSW  2847
CSBV-BJ2012-KF960044.1.pro             TTQWWECADLRLATRENCRAALYQAHGHGSVVYNRFKQQVNKALIKRKIQPIALSW  2847
CSBV—SXnor1-KJ000692.1.pro             TTQWWECADLKLATRENCRAALYQAHGHGSVVYNRFKQQVNKALIKRKIQPIALSW  2847
CSBV-SXYL-KU574662.1.pro               TTQWWECADLKLATRENCRAALYQAHGHGSVVYNRFKQQVNKALIKRKIQPIALSW  2846
AmSBV-Viet6-KM884995.1.pro             TTQWWECADLKLATRENCRAALYQAHGHGSVVYNRFKQQVNKALIKRKIQPIALSW  2846
AcSBV-Viet Nam-KJ959613.1.pro          TTQWWECADLKLATRENCRAALYQAHGHGSVVYNRFKQQVNKALIKRKIQPIALSW  2846
AcSBV-VietBP-KX668139.1.pro            TTQWWECADLKLATRENCRAALYQAHGHGSVVYNRFMQQVNKALIKRKIQPIALSW  2846
AmSBV-Kor2-KP296801.1.pro              TTQWWECADLKLATRENCRAALYQAHGHGSVVYNRFKQQVNKALVKRKIQPIALSW  2846
AmSBV-UK-AF092924.1.pro                TTQWWECADLKLATRENCRAALYQAHGHGSVVYNKFKQQVNQALIKRKIQPIALSW  2845
```
