# Supplementary material for: Genetic and phylogenetic analysis of Chinese sacbrood virus isolates from Apis mellifera
Source: PeerJ. 2019 Nov 14;7:e8003. doi: 10.7717/peerj.8003 (PMC6858986; doi:10.7717/peerj.8003)
Supplement: Supplemental Information 2 — The conserved cysteine protease motif 2249 GXCG 2252 (GACG) and the putative substrate-binding residue 2266 GxHxxG 2271 (GMHFAG) motifs were detected and marked with a red box. [file peerj-07-8003-s002.pdf]

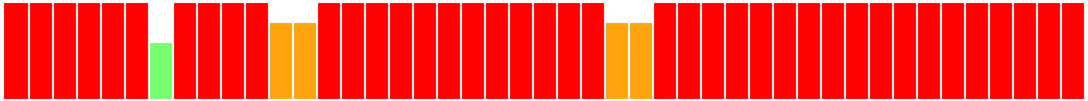

+ Consensus #1		
Consensus #1	GTVFYA.DVIC..YSQQGACGSLCF..RSQRPIVGMHFAGRGEGS	
Majority	GTVFYANDVICYDYSQQGACGSLCFLSRSQRPIVGMHFAGRGEGS	
	2260 2270 2280 2290	
AmCSBV-SDLY.pro	GTVFYANDVICYDYSQQGACGSLCFLSRSQRPIVGMHFAGRGEGS	2276
AcSBV-Kor-HQ322114.1.pro	GTVFYANDVICYDYSQQGACGSLCFLSRSQRPIVGMHFAGRGEGS	2276
AcSBV-Kor4-KP296803.1.pro	GTVFYANDVICYDYSQQGACGSLCFLSRSQRPIVGMHFAGRGEGS	2276
AcSBV-Viet1-KM884990.1.pro	GTVFYANDVICYDYSQQGACGSLCFLSRSQRPIVGMHFAGRGEGS	2276
AcSBV-Viet2- KM884991.1.pro	GTVFYANDVICYDYSQQGACGSLCFLSRSQRPIVGMHFAGRGEGS	2276
AcSBV-Viet3-KM884992.1.pro	GTVFYANDVICYAYSQQGACGSLCFLSRSQRPIVGMHFAGRGEGS	2276
AcSBV-Viet5-KM884994.1.pro	GTVFYANDVICYDYSQQGACGSLCFLSRSQRPIVGMHFAGRGEGS	2276
AcSBV-VietHYnor-KJ959614.1.pro	GTVFYASDVICYDYSQQGACGSLCFLPRSQRPIVGMHFAGRGEGS	2276
AmSBV-Viet4-KM884993.1.pro	GTVFYANDVICHDYSQQGACGSLCFLSRSQRPIVGMHFAGRGEGS	2276
AmSBV-Kor19-JQ390592.1.pro	GTVFYANDVICYDYSQQGACGSLCFLSRSQRPIVGMHFAGRGEGS	2276
CSBV-JL2014-KU574661.1.pro	GTVFYANDVICYDYSQQGACGSLCFLSRSQRPIVGMHFAGRGEGS	2276
AcSBV-VietNA-KX668140.1.pro	GTVFYASDVICYDYSQQGACGSLCFLPRSQRPIVGMHFAGRGEGS	2276
CSBV-FZ-KM495267.1.pro	GTVFYANDVICYDYSQQGACGSLCFLSRSQRPIVGMHFAGRGEGS	2281
CSBV-LN2009-HM237361.1.pro	GTVFYANDVICYDYSQQGACGSLCFLSRSQRPIVGMHFAGRGEGS	2281
CSBV-GD-AF469603.1.pro	GTVFYANDVICYDYSQQGACGSLCFLSRSQRPIVGMHFAGRGEGS	2294
CSBV-BJ2012-KF960044.1.pro	GTVFYASDVICYDYSQQGACGSLCFLSRSQRPIVGMHFAGRGEGS	2294
CSBV—SXnor1-KJ000692.1.pro	GTVFYASDVICYDYSQQGACGSLCFLSRSQRPIVGMHFAGRGEGS	2294
CSBV-SXYL-KU574662.1.pro	GTVFYANDVICYDYSQQGACGSLCFLSRSQRPIVGMHFAGRGEGS	2293
AmSBV-Viet6-KM884995.1.pro	GTVFYASDVICYDYSQQGACGSLCFLSRSQRPIVGMHFAGRGEGS	2293
AcSBV-Viet Nam-KJ959613.1.pro	GTVFYASDVICYDYSQQGACGSLCFLSRSQRPIVGMHFAGRGEGS	2293
AcSBV-VietBP-KX668139.1.pro	GTVFYASDVICYDYSQQGACGSLCFMSRSQRPIVGMHFAGRGEGS	2293
AmSBV-Kor2-KP296801.1.pro	GTVFYANDVICYDYSQQGACGSLCFLSRSQRPIVGMHFAGRGEGS	2293
AmSBV-UK-AF092924.1.pro	GTVFYANDVICYDYSQQGACGSLCFLSRSQRPIVGMHFAGRGEGS	2292
